# Changes Induced by Exposure of the Human Lung to Glass Fiber–Reinforced Plastic

**DOI:** 10.1289/ehp.8676

**Published:** 2006-06-23

**Authors:** Carmelo Abbate, Concetto Giorgianni, Renato Brecciaroli, Giovanni Giacobbe, Chiara Costa, Vittorio Cavallari, Francesca Albiero, Stefania Catania, Maria Antonietta Tringali, Lucia Barbaro Martino, Simona Abbate

**Affiliations:** 1 Department of Social Medicine, Section of Occupational Medicine, Messina University, Messina, Italy; 2 Operative Unit of Pneumology, AOU (Concern Hospital University) Polyclinic G. Martino, Messina, Italy; 3 Operative Unit of Pathology and Ultrastructural Diagnostics, AOU (Concern Hospital University) Polyclinic G. Martino, Messina, Italy; 4 Interdepartmental Centre of Experimental, Environmental and Occupational Toxicology, University of Messina, Messina, Italy

**Keywords:** BAL, biochemical analysis, bronchoalveolar lavage, glass fiber–reinforced plastic, lung, microscopy

## Abstract

The inhalation of glass dusts mixed in resin, generally known as glass fiber–reinforced plastic (GRP), represents a little-studied occupational hazard. The few studies performed have highlighted nonspecific lung disorders in animals and in humans. In the present study we evaluated the alteration of the respiratory system and the pathogenic mechanisms causing the changes in a group of working men employed in different GRP processing operations and exposed to production dusts. The study was conducted on a sample of 29 male subjects whose mean age was 37 years and mean length of service 11 years. All of the subjects were submitted to a clinical check-up, basic tests, and bronchoalveolar lavage (BAL); microscopic studies and biochemical analysis were performed on the BAL fluid. Tests of respiratory function showed a large number of obstructive syndromes; scanning electron microscopy highlighted qualitative and quantitative alterations of the alveolar macrophages; and transmission electron microscopy revealed the presence of electron-dense cytoplasmatic inclusions indicating intense and active phlogosis (external inflammation). Biochemical analyses highlighted an increase in protein content associated with alterations of the lung oxidant/antioxidant homeostasis. Inhalation of GRP, independent of environmental concentration, causes alterations of the cellular and humoral components of pulmonary interstitium; these alterations are identified microscopically as acute alveolitis.

Glass fiber reinforced plastic (GRP) is an extremely versatile structural material composed of unsaturated polyester resin and fiber-glass; objects made from it, obtained by manual or mechanical layering, are characterized by a low specific weight, high resistance to corrosives and atmospheric agents, and low thermal and electrical conductivity. Through a complex chain of events, exposure to fiber-glass contributes to the development of pathologic alterations of the respiratory apparatus for which the etiopathogenesis and evolution are still unclear.

Hesterberg et al. (1993) observed that in rats the exposure to fiberglass does not cause lung fibrosis and/or the statistical increase of lung cancer and mesotheliomas; so they suggest that in humans the exposure to fiberglass dust does not represent a particularly important element in the evolution towards fibrosis and/or lung cancer. Adamson et al. (1995) and McConnell et al. (1999) reached a different conclusion: in studies conducted on animals, they found that glass fibers produced a fibrotic reaction. Dorger et al. (2000) reported a reduction of alveolar macrophage phagocytosis associated with increased production of reactive oxygen species in a study on phagocytic responses in rats and hamsters.

In studies of samples from exposed humans, Chiazze et al. (2002), Enterline et al. (1983), and Riboldi et al. (1999) found inconsistent evidence for a respiratory disease hazard related to exposure to fiberglass. In a case report, Takahashi et al. (1996) described a case of pulmonary fibrosis in a carpenter who had been exposed to glass fibers for 41 years.

Even more complex is the interpretation of the effects caused by exposure to GRP. In fact, the existing studies in the literature do not comprehensively clarify the pathogenetic mechanisms on the basis of the changes that take place (Morisset et al. 1979).

In this study we aimed to further increase the knowledge of alterations to the bronchoalveolar system induced by occupational exposure to GRP and to clarify the pathogenic mechanisms involved.

## Materials and Methods

### Subjects

We excluded subjects from the study based on the following criteria: heavy smokers (> 15 cigarettes/day); subjects with acute or chronic pathologies of the respiratory system; subjects reporting previous occupational exposure that might present a toxic risk for the respiratory system; and subjects with a family and/or personal history of neoplasia and/or respiratory pathologies. The study was conducted on 29 male subjects employed in GRP processing, with a mean age of 37 years and a mean length of service of 11 years. Because all of the workers rotated among the various workstations, according to the company’s organizational structure, the subjects performed the same duties. Exposed subjects had been residents of the area near the production facility since birth. The subjects were divided into groups on the basis of working sector, and environmental measurements were performed for each of them. All of the subjects were volunteers; they were informed about the aim of the study and gave written informed consent before the study began.

All of the subjects came under our observation presenting with an aspecific lung disorder; each subject was subjected to preliminary tests consisting of *a*) a general medical examination with assessment of respiratory symptoms by means of a specially designed questionnaire, *b*) a standard spirometric test, *c*) a test of alveolar-capillary diffusing capacity, *d*) plethysmography, *e*) blood gas analysis, *f* ) standard chest X rays, *g*) electrocardiogram, and *h*) routine blood tests. For all the subjects, we determined urinary mandelic acid using the HPLC method. The HPLC system consisted of a model 616 quaternary pump, a model 600s system controller module, and a 996 diode a ray detector (Agilent Technologies, Waldbrann, Germany).

We then performed a bronchoscopic examination using an Olympus BF-TE2 fiber endoscope (Olympus, Hamburg, Germany).

### Bronchoalveolar lavage (BAL)

After the first medication with atropine, diazepam, and local anaesthetic (Novesine) of the pharynx–larynx–trachea, the subjects were administered five or six loading doses of 30 cc saline solution, heated to 37°C, through the working channel of the fibrobronchoscope; the saline solution was injected into the bronchus of the middle lobe after “wedging” the bronchus or one of its branches (B4 or B5). After each loading dose, the injected material was recovered by suction. The overall quantity administered was 150–180 cc per subject, with an average recovery level of 107 cc (64.85%). The BAL fluid was assessed by the following methods (Clark et al. 2002).

### Microscopy

#### Light microscopy

BAL fluid was centrifuged at 1,200 rpm for 10 min, and the sediment was smeared onto clean glass slides, air-dried, and stained with May-Grünwald-Giemsa (May and Grunwald 1909; Giemsa 1922–1923) and Perl’s Prussian blue (Pearse 1980) for iron deposits. Slides were then evaluated by light microscopy.

#### Transmission electron microscopy

The cells of the sediment were resuspended in phosphate-buffered Karnofsky’s fluid containing 3% glutaraldehyde and 2.5% paraformaldehyde (pH 7.4). Fixation was carried out for 2 hr at 4°C; after several washings in cold phosphate buffer (0.1 M, pH 7.4) the cells were postfixed for 1 hr in 1% phosphate-buffered osmium tetroxide. The cell suspensions were then centrifuged and placed in warm 1% agar solution. After solidification, the agar-included cell suspension was sliced with a razor blade. Blocks, not exceeding 1 mm^3^, were dehydrated in graded ethanols, treated with propylene oxide, and embedded in epoxy resin (Epon 812; Taab Laboratories, Roma, Italy); the blocks were then polymerized at 60°C for 36 hr. Sections (1-μm-thick) were cut with glass knives and stained with 1% toluidine blue in phosphate buffer. Thin sections (40–50 nm) of selected areas were stained with uranyl-acetate and lead citrate in accordance with Reynolds (1963) and observed with a Zeiss CEM 902 electron microscope (Carl Zeiss, Oberkochen, Germany) operating at 80 Kv.

#### Scanning electron microscopy

Cells were fixed in cold modified Karnofsky’s fluid for 3 hr at 4°C, washed in 0.1 M phosphate buffer, and allowed to adhere to a glass cover-slip coated with a 0.1% solution of poly-l-lysine. The cells were subsequently dehydrated using a graded series of ethanols and critical point drying by CO_2_. The specimens were sputter-coated with approximately 30 nm of gold. Evaluations were performed using a field emission scanning electron microscope (Hitachi S-800; Hitachi, Tokyo, Japan) operating at an accelerating voltage of 20 kV.

### Biochemical analyses

Samples were centrifuged at 1.5 × *g* for 10 min at 4°C, and the resulting supernatant was stored at –70°C until use. We determined the protein content of recovered BAL fluids according to the method of Bradford (1976).

#### Antioxidant enzymes and reduced glu-tathione (GSH)

We quantified levels of antioxidant enzymes in the BAL fluid of exposed and control subjects, which had been previously concentrated 10-fold by ultra-filtration using the Ultrafree-CL, NMWL 5,000, centrifugal filter unit (Millipore, Billerica, MA, USA).

We measured catalase (CAT) activity following the method of Aebi (1984); CAT units were calculated using the formula U/mL = *k*_15”_/mL, where *k*_15”_ = 0.153 (log A_0_/A_15”_); A_0_ and A_15”_ corresponded to absorbance at 240 nm at times 0 and 15 sec, respectively.

Superoxide dismutase (SOD) activity was determined according to Misra and Fridovich (1972) with some modifications. Samples were diluted as for the CAT assay and mixed with 0.04 M phosphate buffer containing 0.08 M EDTA and 0.8 mM *N*,*N*,*N*′,*N*′-tetramethylethylenediamine (pH 10). Quercetin (0.44 mM in dimethylformamide) was added, and the decrease in absorbance was immediately read at 406 nm for 10 min at 25°C. SOD activity was extrapolated from a calibration curve.

GSH content was measured according to Dietz and Rubinstein (1972). BAL samples were diluted 1:4 with reaction mixture, and results were extrapolated from a calibration curve.

#### Lipid peroxidation

We measured thio-barbituric acid–reacting substances by diluting BAL samples 1:3 with the reaction mixture described by Buege and Aust (1978). Results are expressed as malondialdehyde (MDA) production, representative of lipid peroxidation products, calculated using a 1,1,3,3-tetraethoxypropane standard curve.

We compared the results from the subjects with those from a control sample of 28 males. The controls were homogenous in terms of age (mean ± SD, 35.4 ± 11.1 years), and were selected based on the same criteria as subjects [i.e., they were not heavy smokers (> 15 cigarettes/day)], did not have acute or chronic pathologies of the respiratory system, had no previous occupational exposure to toxic substances that affect the respiratory system, and did not have family and/or personal history of neoplasia and/or respiratory pathologies. The controls were residents of the same area as the exposed subjects, had not been exposed to GRP, and had been subjected to BAL for diagnostic purposes (4 for the extraction of a foreign body, 12 for persistent cough without expectoration, 3 for hemophthysis, 9 for suspected fibrosis).

### Exposure assessment

The study subjects were employed by a firm producing GRP tubes; during the production process, employees apply raw materials (e.g., a thermosetting resin based on styrene and fiberglass) to a spindle to form tubes. Accessory operations are also performed on the finished tubes, such as cutting for the production of special pieces, finishing, and repair by means of manual resin application.

We assessed the exposure of the workers through a series of environmental measurements performed in the year 2002. The environmental sampling of styrene and total and respirable dusts was performed using constant flow personal samplers (PERSONAL Basic; Aquaria, Milano, Italy). For styrene, we used sorbent tubes, the aspiration flows and times, and the analytical procedure for the gas chromatographic quantitative dosing as described in National Institute for Occupational Safety and Health (NIOSH) method 1501 (NIOSH 1994c). All samples were collected approximately 1.6 m above the floor. For total and respirable dusts, the use of filters, aspiration flows and times, and the analytical procedure for gravimetric quantitative dosing were in compliance with NIOSH Method 500 (NIOSH 1994a) and Method 600 (NIOSH 1994b), respectively.

Table 1 shows the results of environmental sampling. The concentrations of styrene and total dusts turned out to be lower than the threshold limit value (TLV) – time-weighted average (TWA); the values of breathed dust, reported for the operations of sleeve production and resin preparation, were > 3 mg/m^3^ (TLV –TWA). Examination of the sampled air under the electron scanning microscope highlighted the presence of fragments of glass fibers.

## Results

The anamnestic and clinical data for the exposed subjects highlighted mild dyspnea under stress, and mild asthenia not associated with other chemical signs in all the subjects. Twenty-eight percent of the exposed subjects reported coughing in the previous 6 months, with expectoration reported in 14% of these.

In the control group, 42% of the subjects reported persistent coughing without expectoration in the previous 6 months, and 10% reported hemophthysis.

In exposed subjects, the results of the basic tests (spirometry, CO_2_ diffusing capacity, plethysmography) were negative, with the exception of the spirometry test. In that test, 28% of the exposed subjects showed a mild respiratory syndrome of a bronchiolar-obstructive nature. The results of the basic tests on the control subjects were all normal.

Mandelic acid levels, used as an indicator of exposure, were < 5 mg/L (negative result) in all subjects tested.

### Counts of BAL cells

The number of BAL cells in exposed subjects was higher than in control subjects, as shown in Table 2.

### Light microscopy

**Figure 1:**
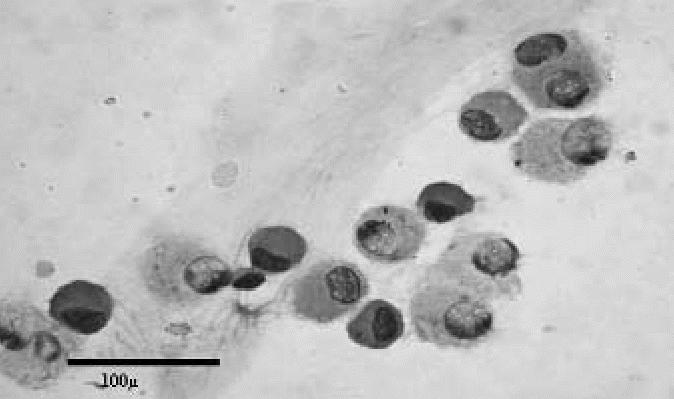
Cytology of BAL sample from an exposed subject showing a predominance of small and medium-sized macrophages (May-Grunwald-Giemsa). Bar = 100 μm.

**Figure 2:**
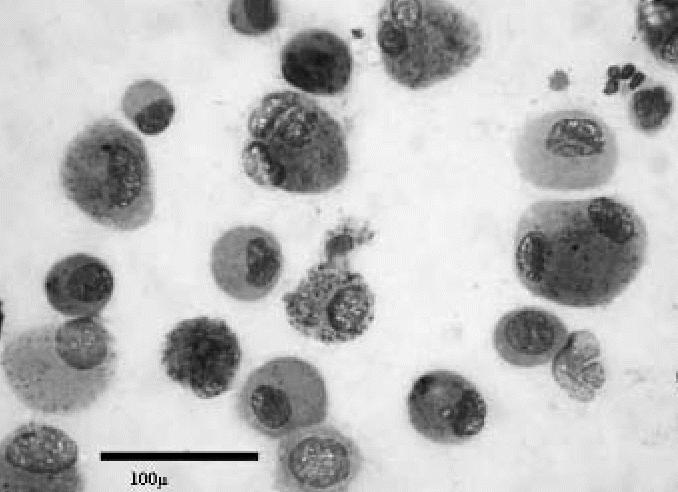
Cytology of BAL sample from an exposed subject showing a predominance of large macrophages (May-Grunwald-Giemsa). Bar = 100 μm.

The smears stained with Giemsa were examined using a light microscope. We found a significant increase of alveolar macrophages in all exposed subjects compared with the controls (*t* = 2.112; *p* = 0.039), with a mean ± SD of 87.9 ± 5.04 expressed as a percentage of the total cell count (mean 6.2 × 10^6^cells/mL) (Table 1). The macrophages showed a variable amount of cytoplasmic inclusions and considerable variability in size (Figures 1 and 2). In all cases, the cytologic findings suggested an abnormal increase of alveolar macrophages.

### Scanning electron microscopy

**Figure 3:**
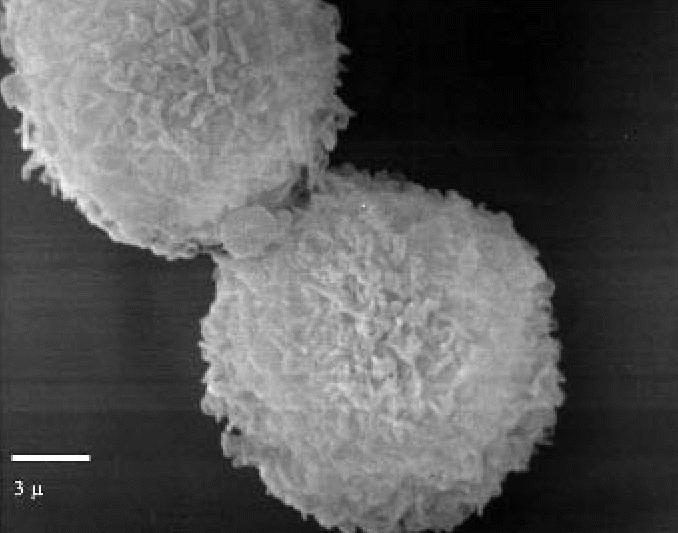
Scanning electron micrograph showing the surface pattern of small macrophages (exposed subject). Note the fine philopodial expansions. Bar = 3 μm.

**Figure 4:**
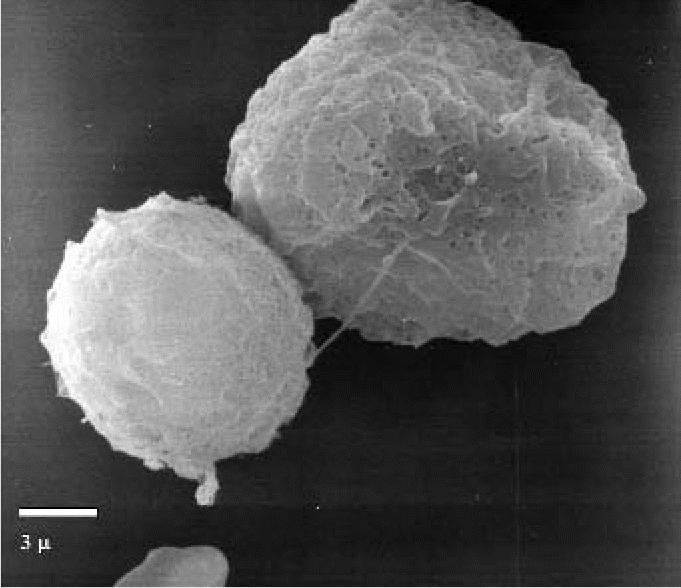
Scanning electron micrograph showing the “smooth” surface pattern of large macrophages (exposed subject). Bar = 3 μm.

Most of the specimens showed an absolute predominance of macrophages, and there were two main surface patterns. The first pattern, corresponding to a predominance of medium-sized macrophages, involved complex surfaces with abundant small philopodial expansions (Figure 3). The second pattern was mainly associated with a predominance of large macrophages and consisted of relatively simple surface patterns with short and large expansions (Figure 4).

### Transmission electron microscopy

In the control groups the BAL samples contained a significant percentage of epithelial cells displaying well-preserved cilia. Most of the alveolar macrophages were medium-sized, lamellar inclusions containing abundant cytoplasm, which is consistent with surfactant material.

**Figure 5:**
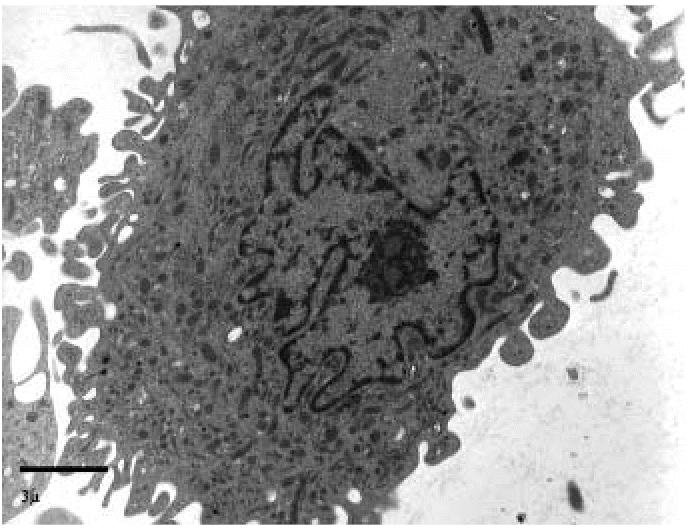
Transmission electron micrograph showing small macrophages, the cytoplasm of which contains prevalently primary lysosomes (exposed subject). Bar = 3 μm.

In all exposed subjects, the BAL samples were composed predominantly of alveolar macrophages, with a significant variability in size, shape, and organization of the subcellular compartments. Small macrophages had convoluted nuclei with marginated chromatin, relatively evident nucleoli, and abundant cytoplasmic organelles. In particular, rough endoplasmic reticulum and small primary lysosomes were predominant (Figure 5); the cell borders showed long filopodial expansions with a “hairy” appearance.

**Figure 6:**
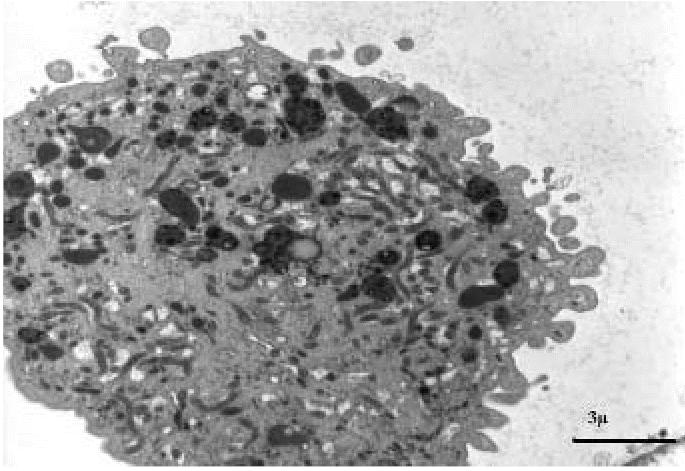
Transmission electron micrograph showing medium-sized macrophages containing both large complex inclusions and several primary lysosomes (exposed subject). Bar = 3 μm.

Medium-sized macrophages had the same nuclear features as small macrophages. Cytoplasms contained a variable amount of electron-dense inclusions, ranging from relatively small dyshomogeneous lysosomes to large irregularly dense inclusions (Figure 6). A relatively small number of medium-sized macrophages contained round lamellar inclusions consistent with surfactant material.

**Figure 7:**
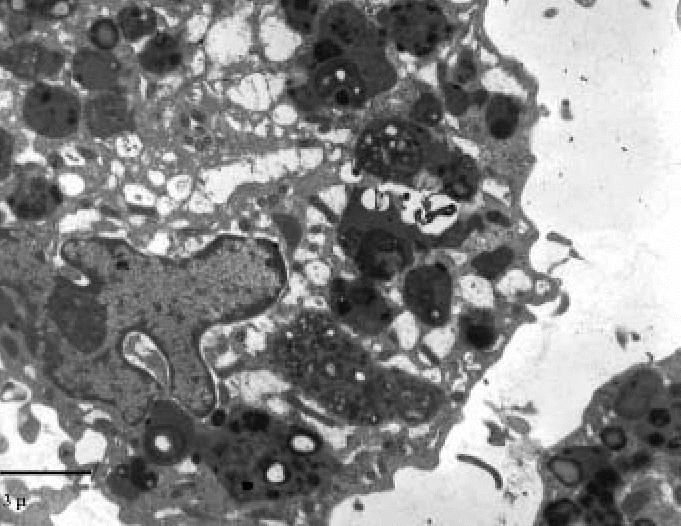
Transmission electron micrograph showing large macrophages containing prevalently complex inclusions with very dense granular deposits consistent with iron particles (exposed subject). Bar = 3 μm.

Large macrophages showed convoluted nuclei with dense chromatin; nucleoli were not evident. Cytoplasms were filled with dense inclusions exhibiting an admixture of components with different electron density and extremely dense granular particles, consistent with iron deposits demonstrated in light microscopy (Figure 7).

Biochemical analyses performed on BAL samples are summarized in Table 3.

GRP-exposed subjects presented a significant increase of total protein concentrations in the lavage fluid compared with controls. Consequently, as proteins behaved like a dependent rather than an independent variable, their concentrations and those of the other biochemical parameters were normalized on the basis of total BAL volume retrieved in each subject.

GSH showed a significant decrease in exposed subjects, suggesting modifications in the lung antioxidant status, as confirmed by the induction of antioxidant enzymes. CAT showed a significant increase of about 3-fold.

SOD, despite a 5-fold increase, did not reach statistical significance; this may be explained by the high variability of this parameter.

Finally, MDA production, representative of lipid peroxidation, was also enhanced in GRP-exposed subjects compared with controls.

## Discussion

In the present study we observed that exposure to GRP causes an inflammatory alteration of the deep lung with few clinical symptoms, mild dyspnea under stress, and mild dysfunctions of a bronchiolar-obstructive nature, accompanied by characteristic alterations of the BAL fluid, both in terms of cellularity and biochemical specifications.

Literature on this subject, mainly experimental, reports that occupational exposure to fiberglass causes the development of an inflammatory process affecting the lungs. In experimental studies on animals (rats and hamsters) and *in vitro*, Dorger et al. (2000, 2001) highlighted the role that alveolar macrophages play in pulmonary clearance. They also argued that the persistence of the glass fibers in the bronchial tree is correlated to the process of macrophagic phagocytosis and to the production of toxic oxygen radicals and SOD, which contribute to the inflammatory process. Other authors reported that the prolonged exposure to the inhalation of fiberglass alters the surface morphology of the alveolar macrophages. For example, in a study on alveolar macrophages observed before and after rats were exposed to fiberglass, Luoto et al. (1994) reported changes in the conformation of the macrophagic membrane characterized by cytoplasmatic expansions about 20 μm long, which increase in direct proportion to the increase in cytoplasmatic inclusions. Morisset et al. (1979), in an experimental study in rats, reported that exposure to fiberglass and styrene induced an alteration of the cells of the bronchiolar epithelium with a predominance of apocrine cells.

The results obtained in our exposed subjects showed increased macrophagic cellularity and increased oxidative activity in the BAL. Under scanning electron microscopy, the alveolar macrophages were significantly altered, both in terms of size and of the characteristics of the cytoplasmatic surface, displaying two cell types: macrophages of medium size with narrow filopodial expansions and very large macrophages with short and wide expansions on the cellular surface. Under transmission electron microscopy, electron-dense cytoplasmatic inclusions were present, indicating intense and active phlogosis.

The results of biochemical analysis con-firmed those of microscopy; in fact, the BAL fluid from the exposed subjects displayed increased protein content associated with glutathione depletion, and induction of CAT and SOD—conditions that indicate a modification of the antioxidant state of the lung.

The trend of the data on the biochemical alterations of BAL fluid is similar to those reported in experimental studies (Brown et al. 2000; Topinka et al. 2006). These data highlight the occurrence of oxidative stress accompanied by an enzymatic alteration, characterized by glutathione depletion in the lung tissue and alteration of the cellular cytoplasmatic components. In a study of the effects of the inhalation of styrene on the respiratory system in rats, Coccini et al. (1997) reported that the alterations are dose-dependent and more severe for prolonged exposure. Laffon et al. (2002) believe that these anomalies are always associated with glutathione depletion.

The biochemical modifications observed in our sample confirmed the hypothesis that oxidative stress activates the inflammatory process in the lung. In fact, in all of the GRP-exposed subjects, we found an increase in the proteins CAT and SOD associated with a reduction in glutathione. This indicates the activation of an inflammatory process of the pulmonary parenchyma, which occurs by means of a direct mechanism with the activation of the alveolar macrophages and by indirect mechanisms characterized by an oxidant/antioxidant imbalance.

The results of the present study show that in GRP-exposed subjects there are characteristic alterations in the enzymatic content and cellularity of the BAL fluid. In addition to quantity, the alterations regard the quality of the cells, in particular the size, as we observed by examining the BAL smears under the light microscope.

By way of confirmation of our data there are experimental studies that confirm the development of a pulmonary inflammatory process following exposure to fiberglass.

In conclusion, our results show, for the first time in humans, that fiberglass—independent of the environmental concentration—causes an alteration of the cellular and enzymatic components of the deep lung. We microscopically identified these alterations with the acute alveolitis.

## Figures and Tables

**Table 1:** Concentrations (mg/m^3^) of styrene, total dusts, and respirable fractions.

Pollutant	Sampling station	Airborne concentration	TLV – TWA
Styrene	Polymerization	0.458	
	Gluing	0.002	215
	Sleeves	0.264	
Total dusts	Gluing	6.621	10
	Grinding	7.650	
Respirable fraction	Sleeves	4.166	
	Mastic	4.042	3
	Grinding	0.901	

**Table 2:** Cellular population (mean ± SD) of the exposed and control groups.

Cell type (cells/mL × 10^6^)	Exposed	Control
Total	6.2 ± 5.38	5.04 ± 2.49
Macrophages	87.9 ± 5.04	85.25 ± 4.4
Lymphocytes	10.4 ± 4.63	14.35 ± 4.51
Granulocytes		
Neutrophils	1.7 ± 2.12	0.15 ± 0.39
Eosinophils	0	0.75

**Table 3:** Results (mean ± SD) and associated *p*-values (significant difference) for total proteins, GSH, SOD, CAT, and MDA in BAL based on total volume retrieved.

Subjects	Proteins (μg/mL)	GSH (nmol/mL)	CAT (U/mL)	SOD (U/mL)	MDA (nmol/mL)
Controls (*n* = 28)	51.84 ±10.73	1.206 ± 0.196	21.62 ± 5.46	50.55 ± 26.48	0.071 ± 0.012
Exposed (*n* = 28)	150.8 ± 36.67	0.526 ± 0.147	66.00 ± 34.55	247.1 ± 112.7	0.167 ± 0.040
	*p* > 0.001	*p* > 0.001	*p* > 0.001	NS	NS

NS, not significant.
